# Beyond Sustainable: Geo-Adaptive Design of Carbon-Based Adsorbents Through Aligning Pesticide Remediation with Regional Agricultural Practices and Food Safety Needs

**DOI:** 10.3390/foods15061110

**Published:** 2026-03-23

**Authors:** Tamara Lazarević-Pašti, Igor A. Pašti

**Affiliations:** 1 VINČA Institute of Nuclear Sciences—National Institute of the Republic of Serbia, University of Belgrade, Mike Petrovica Alasa 12–14, 11000 Belgrade, Serbia; tamara@vin.bg.ac.rs; 2 Faculty of Physical Chemistry, University of Belgrade, Studentski Trg 12–16, 11158 Belgrade, Serbia; 3 Serbian Academy of Sciences and Arts, Kneza Mihaila 35, 11000 Belgrade, Serbia

**Keywords:** pesticide residues, biomass valorization, adsorption mechanisms, microporous carbon, decentralized mitigation strategies, circular economy

## Abstract

The persistence of pesticide residues in food and water poses a significant challenge to global food safety, particularly under the pressures of intensive agriculture and climate variability. Despite significant progress in developing adsorbent materials for pesticide remediation, most approaches remain chemically optimized but geographically blind. This review introduces the concept of geo-adaptive design of carbon-based adsorbents, emphasizing that remediation materials should be tailored to the regional profiles of pesticide use, environmental conditions, and available biomass precursors. Pesticide contamination patterns vary widely across climates and agricultural systems, resulting in distinct chemical signatures that determine adsorption behavior. Simultaneously, locally abundant agro-industrial byproducts, such as walnut shells, rice husks, olive stones, or fruit pomace, offer sustainable carbon sources for region-specific materials. By correlating pesticide structure, adsorbent surface chemistry, and environmental parameters, geo-adaptive materials can be designed to maximize efficiency, selectivity, and sustainability in environmental remediation contexts, including the treatment of pesticide-contaminated soils and water streams. In addition, these materials may be integrated into food processing and packaging systems, where they can function as localized, low-cost mitigation strategies aligned with circular economy principles. The review highlights how regionally optimized carbon materials could connect advances in environmental remediation with the practical needs of food technology, leading toward food safety strategies that are both globally relevant and locally adaptable.

## 1. Introduction

The widespread use of pesticides in modern agriculture has contributed significantly to crop protection and food security [1]. Still, it has also led to the persistent presence of pesticide residues in food products and associated processing streams [2]. Residues from pre-harvest treatments, post-harvest preservation, and contaminated irrigation water remain a major concern for food safety, particularly when conventional washing or processing steps are insufficient to fully remove chemically stable compounds [3]. Climate variability, intensification of agriculture, and expanding global food trade further complicate this issue by altering pesticide degradation pathways and redistributing contamination risks across regions [4].

Carbon-based adsorbents, including activated carbons [5], biochars [6], and graphene-derived materials [7,8], have been extensively investigated as effective tools for pesticide removal from water and food-related matrices [9,10,11]. Owing to their high surface area, well-defined surface functional groups, and strong affinity toward a wide range of organic molecules [12,13,14,15], these materials are often proposed as universal solutions for pesticide remediation. However, most existing studies focus on maximizing adsorption capacity under standardized laboratory conditions [9,10,11,16], while paying limited attention to the geographical context in which pesticide contamination actually occurs. In practice, pesticide use patterns are strongly region-dependent, shaped by local crop types, climatic conditions, regulatory frameworks, and agricultural practices [17,18]. As a result, the chemical nature of dominant pesticide residues, such as polarity, functional groups, persistence, and toxicological relevance, varies substantially between regions. At the same time, the availability of biomass resources suitable for producing carbon-based adsorbents also differs geographically, offering region-specific opportunities for sustainable material design that are rarely considered in remediation strategies [19,20].

In this paper, we propose the concept of geo-adaptive design of carbon-based adsorbents to align remediation materials with regional pesticide profiles, environmental conditions, and locally available precursors. Rather than seeking universally optimal adsorbents, a geo-adaptive approach emphasizes context-driven material selection and design to improve efficiency, selectivity, and sustainability in food safety applications. This review examines how the chemical properties of regionally dominant pesticides and local agricultural conditions can inform the rational selection and design of carbon-based adsorbents to mitigate pesticide residues in environmental matrices and in food processing and related systems.

This work is structured as a narrative review that develops a conceptual framework for geo-adaptive design of carbon-based adsorbents. The literature was identified through searches in major scientific databases, including Web of Science, Scopus, and Google Scholar, using combinations of keywords such as carbon adsorbents, biochar, pesticide adsorption, food safety, and biomass-derived carbon materials. Priority was given to peer-reviewed research articles and recent review papers addressing adsorption mechanisms, biomass-derived carbon materials, regional pesticide usage patterns, and applications relevant to food systems.

## 2. Global Diversity of Pesticide Use and Residues

Patterns of pesticide use vary noticeably across geographical regions [21,22,23,24,25,26], reflecting differences in dominant crops, climatic conditions, pest pressure, and regulatory frameworks. These regional differences directly influence the types and concentrations of pesticide residues detected in food commodities and associated water streams [4], thereby shaping food safety challenges at the local scale. While global monitoring programs provide valuable aggregated data, they often mask substantial regional heterogeneity that is critical for designing effective mitigation strategies.

**Figure 1:**
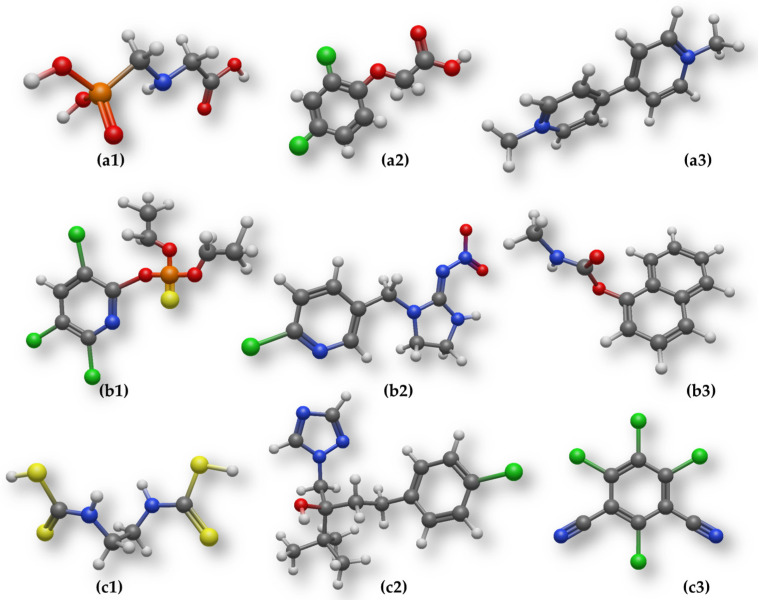
Representative molecular structures of selected pesticides covering the major chemical classes of herbicides, insecticides, and fungicides. The set includes herbicides ((**a1**)—glyphosate, (**a2**)—2,4-D, (**a3**)—paraquat), insecticides ((**b1**)—chlorpyrifos, (**b2**)—imidacloprid, (**b3**)—carbaryl), and fungicides ((**c1**)—mancozeb, (**c2**)—tebuconazole, (**c3**)—chlorothalonil). These compounds were selected as illustrative examples of widely used pesticide classes across major agricultural regions and to represent structurally diverse molecules relevant to adsorption behavior and geo-adaptive remediation strategies (grey—carbon, white—hydrogen, red—oxygen, blue—nitrogen, green—chlorine, yellow—sulfur, orange—phosphorus).

In Mediterranean regions, including large parts of Southern Europe, organophosphates and certain triazole fungicides have historically been widely applied in fruit, vegetable, and vineyard production [24,27,28]. Although regulatory restrictions have reduced the use of some legacy compounds, their persistence in soils and irrigation systems means that residues can still be detected in raw agricultural products and processing waters [29]. In contrast, intensive rice, vegetable, and orchard farming in many Asian regions has been associated with extensive use of neonicotinoids and carbamate insecticides [30,31,32,33,34,35], compounds characterized by higher polarity and water solubility, which facilitates their transfer into aqueous food-processing streams. In North and South America, large-scale cereal and oilseed production has driven the predominant use of herbicides [21,26,36,37], particularly glyphosate-based formulations, leading to a distinct residue profile that differs fundamentally from that of insecticide-dominated systems. Table 1 presents representative pesticide active substances, grouped by functional class, across major regulatory regions to illustrate geographic differences in pesticide use relevant to food safety, while Figure 1 presents the structure of several representative pesticides from different classes.

The authorization and use of pesticides are governed by region-specific regulatory frameworks that play a central role in shaping pesticide application patterns and, consequently, food-safety-relevant residue profiles. In the United States, pesticide regulation is overseen by the U.S. Environmental Protection Agency (EPA) [38], which evaluates active substances and registers pesticide products under the Federal Insecticide, Fungicide, and Rodenticide Act [39]. Approval is use-specific and subject to periodic re-evaluation, leading to the continued use of certain legacy compounds alongside newer formulations. Within the European Union, pesticide regulation follows a hazard-based approach coordinated by the European Food Safety Authority (EFSA) [40], with active substances approved at the EU level and Maximum Residue Levels (MRL) established for food commodities. This framework has led to the withdrawal of several organophosphates and herbicides [41], producing a residue landscape dominated by selected fungicides and a limited number of insecticides. In China, pesticide registration and management are conducted at the national level by the Ministry of Agriculture and Rural Affairs (MARA) [42], with extensive approval of insecticides and herbicides to support intensive crop production systems. Similarly, in India, pesticide use is regulated by the Central Insecticides Board and Registration Committee (CIBRC) [43], where a broad spectrum of active substances remains registered, reflecting the country’s diverse agro-climatic conditions and pest pressures. In South America, Brazil serves as a representative regulatory system due to its scale and agricultural output. Pesticide registration is managed by the Department of Agriculture, Livestock and Food Supply (MAPA) [44] using the AGROFIT system [45] with health-related assessments performed by the National Health Surveillance Agency (Anvisa) [46]. As a result, Brazil maintains authorization for several herbicides and insecticides that are no longer permitted in the EU, contributing to a distinct regional pesticide profile with direct implications for food safety and remediation needs.

**Figure 2:**
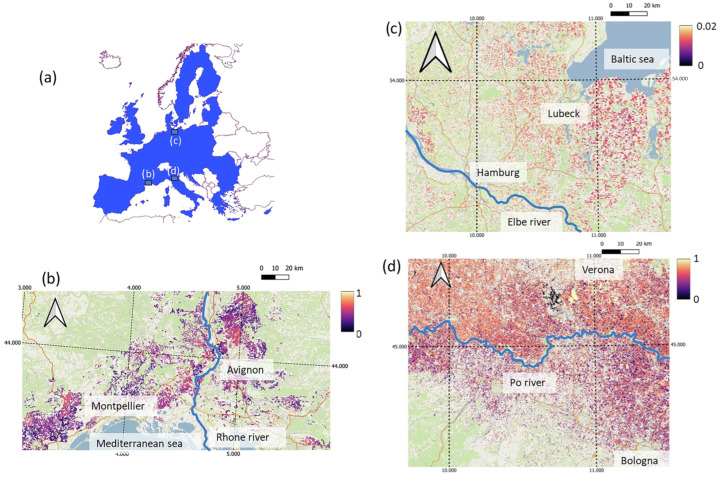
Study area (blue shade in (**a**)) and details of sample maps of median application rates: (**b**) fungicide Mancozeb in southern France, (**c**) insecticide Dimethoate in northern Germany, (**d**) herbicide Glyphosate in northern Italy. All application rates are given in [kg/ha]. Background information is obtained from OpenStreetMap layers, and labels on map boundaries indicate longitude and latitude (WGS84 coordinates). Reproduced from [52]. This article is licensed under a Creative Commons Attribution 4.0 International License [53], without any modifications.

Climatic conditions further modulate pesticide fate and persistence [4]. Temperature, solar irradiation, rainfall patterns, and soil moisture influence degradation pathways, leaching behavior, and bioavailability of pesticide residues [47,48,49]. For example, higher temperatures and intense solar exposure may accelerate photodegradation of certain compounds, while simultaneously increasing volatilization and off-site transport [50,51]. Conversely, in cooler or more humid climates, reduced degradation rates and stronger sorption to organic matter can promote residue accumulation. These climate-driven effects are increasingly altered by climate change, introducing additional uncertainty into regional contamination patterns. It underscores the need for precise monitoring of local distributions of pesticides and residues. Unfortunately, such studies are rare because they require significant resources and yield data with time-limited validity due to the variability of numerous factors that affect the distribution of these chemicals; however, some can be found in the literature. For example, Porta et al. reported pesticide application rate maps for the European Union at a 250 m spatial resolution [52], as shown in Figure 2. Such data are highly useful for planning exposure mitigation and remediation strategies, and their role in the geo-adaptive adsorbent design concept will be discussed later on.

From a food safety perspective, this geographic variability means that food products entering processing and distribution chains are exposed to fundamentally different pesticide chemistries depending on their origin. Residues may differ not only in concentration, but also in molecular structure, polarity, stability, and toxicological relevance [54,55]. Nevertheless, many remediation approaches implicitly assume a uniform contaminant profile, thus applying the same adsorbent materials or treatment strategies across regions with vastly different agricultural realities. Recognizing the global diversity of pesticide use and residue profiles is therefore a prerequisite for moving beyond generic mitigation strategies. A geographically informed understanding of dominant pesticide classes, combined with regional environmental conditions, provides the foundation for designing adsorption-based remediation approaches that are better aligned with actual food safety needs rather than idealized laboratory scenarios.

## 3. Matching Adsorbent Chemistry to Regional Contaminant Profiles

**Figure 3:**
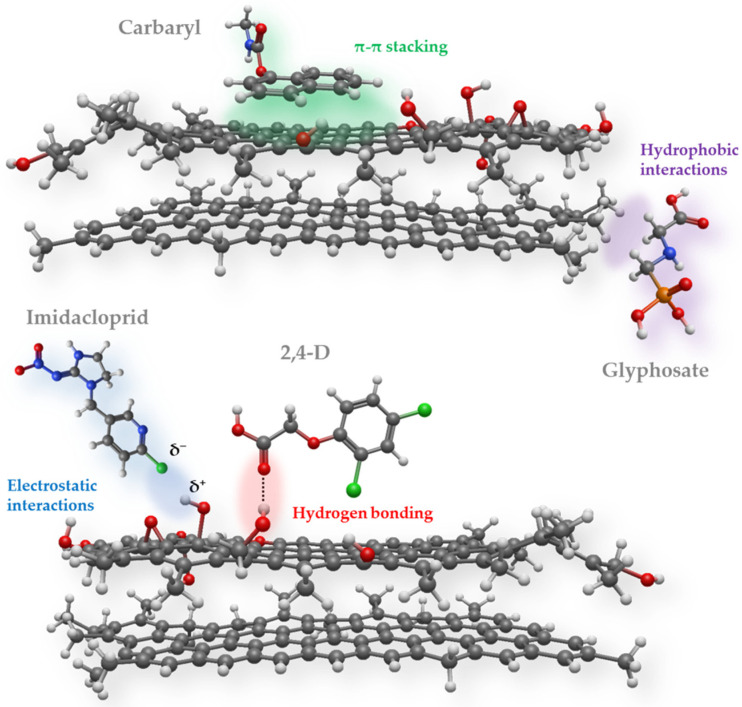
Schematic representation of different types of pesticide interactions with carbon surfaces and the surface functional groups (grey—carbon, white—hydrogen, red—oxygen, blue—nitrogen, green—chlorine, orange—phosphorus).

The effectiveness of carbon-based adsorbents in pesticide remediation is governed not only by general textural properties, such as surface area and porosity, but more critically by the compatibility between adsorbent surface features and the chemical characteristics of target pesticide residues [9,10,11]. As illustrated in Table 1, dominant pesticide classes may differ across regions, indicating that adsorption strategies optimized for one contaminant profile may not necessarily be optimal in other geographic contexts. Herbicide-dominated agricultural systems, such as those prevalent in the United States and Brazil, are largely characterized by highly polar or ionic compounds, including phosphonate- and phenoxy-based herbicides [56]. These molecules interact weakly with nonfunctionalized graphitic surfaces, which are essentially nonpolar, hydrophobic, and chemically inert. Thus, their effective removal typically requires adsorbents with oxygenated or charged surface groups capable of hydrogen-bonding or electrostatic interactions [57,58,59] (Figure 3). In such contexts, carbon materials with a higher density of acidic or basic functional groups, rather than maximized hydrophobicity, are more likely to achieve relevant removal efficiencies under food-processing conditions. In contrast, regions where insecticide use is dominated by organophosphates and carbamates, as observed in parts of Asia and India [60,61], present a mixed adsorption challenge. These compounds often combine moderate polarity with aromatic or heterocyclic moieties, enabling multiple interaction pathways, including hydrogen bonding, dipole–dipole interactions, and π-π stacking [62]. Adsorbents optimized for these systems must therefore balance surface functionality with accessible aromatic domains, emphasizing the importance of surface heterogeneity over uniform chemical modification. Fungicide-intensive regions, particularly within European and Mediterranean agricultural systems, are frequently associated with triazole and chlorinated fungicides characterized by aromatic rings, moderate hydrophobicity, and high chemical stability [63,64,65]. For these compounds, adsorption is largely driven by π-π interactions and hydrophobic affinity, favouring carbon materials with extended conjugated domains and well-preserved graphitic regions [65,66]. Excessive oxidation or highly polar surfaces may be counterproductive in such cases, despite their benefits for other contaminant classes. Figure 3 schematically presents the most important types of interactions between pesticides and carbon surfaces with different chemistries.

**Figure 4:**
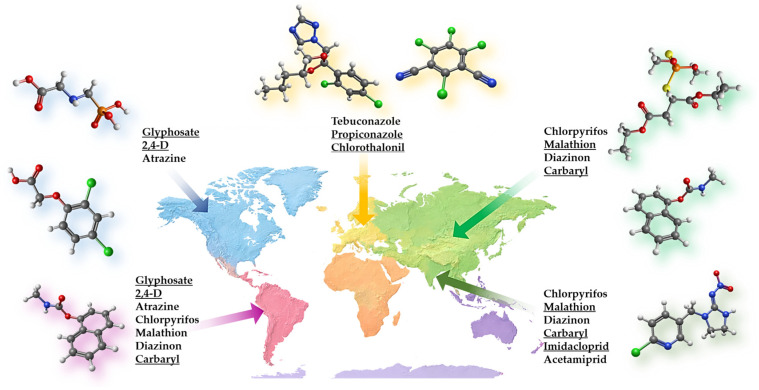
Regional usage of most common pesticides (the structures are displayed for the underlined ones, grey—carbon, white—hydrogen, red—oxygen, blue—nitrogen, green—chlorine, yellow—sulfur, orange—phosphorus).

Beyond pesticide structure, regional environmental conditions further modulate adsorption behaviour [16]. pH, ionic strength, natural organic matter, and coexisting ions in irrigation or processing waters can significantly alter both pesticide speciation and adsorbent surface charge. As these parameters are closely linked to local climate and water sources, geo-adaptive design requires that adsorbent chemistry be considered alongside realistic environmental matrices rather than idealized laboratory conditions. These considerations emphasize that adsorption performance should be evaluated with respect to the dominant pesticide chemistries encountered in a given region and under conditions that simulate the local environment. Therefore, a geo-adaptive approach should prioritize the rational matching of pesticide class, surface functionality, and environmental conditions over the pursuit of universally high adsorption capacities. Such alignment is particularly critical in food safety applications, where mitigation strategies must operate effectively under mild processing conditions, in complex matrices, and with minimal impact on product quality. Table 2 and Figure 4 explain how regional pesticide use patterns inform material design criteria by linking dominant pesticide classes to their key chemical features and the corresponding surface characteristics required for effective adsorption. Regional pesticide usage patterns are closely linked to the dominant crops cultivated in each agricultural system. Herbicide-intensive regions such as North and South America are largely associated with large-scale cereal and oilseed production, including maize, wheat, and soybean systems, where weed management represents a major agronomic challenge. In contrast, regions characterized by intensive fruit, vegetable, and orchard production, particularly in parts of Europe and the Mediterranean, tend to rely more strongly on fungicides and insecticides. Similarly, rice- and vegetable-based agricultural systems in many Asian regions are associated with extensive use of neonicotinoid and carbamate insecticides. These crop–pesticide relationships provide important context for interpreting the regional pesticide profiles illustrated in Figure 4 and highlight the relevance of geo-adaptive adsorbent design for food-related contamination scenarios.

## 4. Utilizing Regional Biomass as Precursors for Sustainable Carbon-Based Adsorbents

The geo-adaptive design of adsorption materials is not limited to matching surface chemistry with regional pesticide profiles. It also extends to the selection of appropriate precursor materials. Across major agricultural regions, large volumes of agro-industrial residues are generated as byproducts of food production and processing [67]. These materials represent underutilized carbon sources that can be transformed into functional adsorbents, offering both environmental and economic advantages within region-specific food systems. The chemical composition of biomass precursors, including lignin, cellulose, hemicellulose, proteins, and inorganic minerals, strongly influences the structure and surface properties of the resulting carbon materials [68,69,70]. As a consequence, locally available biomass can yield carbon materials with characteristic textural features and surface functionalities that tend to favor the adsorption of pesticide classes prevalent in the same agricultural systems from which the biomass originates. This apparent compatibility does not arise from geographic coincidence but from shared agro-ecological and processing contexts that simultaneously shape crop-residue and pesticide-application profiles. The link between precursor composition and adsorbent properties provides a natural foundation for geo-adaptive material design.

In South-eastern and Central Europe, walnut shells and related nut-processing residues are abundant byproducts of food and oil industries [71,72,73]. Their high lignin content promotes the formation of aromatic carbon frameworks upon carbonization [74], yielding materials with pronounced π-conjugated domains and moderate surface polarity. These features are compatible with the adsorption of organophosphate insecticides and triazole fungicides, which are frequently encountered in Balkan and Southern European agricultural systems [6,75,76,77]. In addition to their adsorption performance, the local availability of walnut-derived residues supports low-cost material production while reducing the environmental footprint associated with transportation and centralized processing. Mediterranean regions of Southern Europe also generate substantial quantities of olive stones, pomace, and other fruit-processing residues [78,79,80,81]. Carbons derived from these precursors typically display a predominantly microporous structure with moderate surface polarity [82,83,84,85], features that are well suited to the adsorption of small- and medium-sized pesticide molecules commonly applied in fruit, vegetable, and vineyard production [86,87,88]. The valorization of olive- and fruit-derived residues is therefore closely aligned with circular economy strategies already embedded in Mediterranean food-producing regions.

In the same Mediterranean agricultural contexts, citrus peels and processing residues constitute an abundant, seasonally stable biomass stream directly linked to fruit and juice production chains [79]. Citrus-derived biomass, rich in pectin, cellulose, and oxygen-containing functional groups [89,90], can yield carbon materials with a relatively high density of surface oxygen functionalities after carbonization or activation [91,92]. Such surface characteristics favor interactions with moderately polar pesticide residues associated with fruit production, including selected organophosphates and fungicides [93,94]. Importantly, citrus-based carbons are particularly attractive for food-related applications due to their low cost, local availability, and seamless integration into existing citrus-processing workflows, thereby reinforcing the geo-adaptive design paradigm for Mediterranean food systems.

Across East and Southeast Asia, rice cultivation dominates agricultural landscapes and generates vast quantities of rice husks and straw as byproducts of milling and processing [95]. Rice husk-derived biomass is characterized by a unique combination of lignocellulosic components and a high intrinsic silica content [96,97], which strongly influences the structure of the resulting carbon materials [98,99,100,101]. Upon carbonization or activation, rice husk-based carbons often develop hierarchical pore architectures and relatively polar surface features [102,103,104], enhancing accessibility and interaction with water-soluble pesticide residues. These characteristics are particularly relevant in regions where neonicotinoid and carbamate insecticides are widely applied in rice and vegetables [105,106,107]. The high polarity and aqueous mobility of such compounds favor adsorption mechanisms that rely on surface functional groups and accessible pore networks rather than purely hydrophobic interactions [108]. In this context, rice husk-derived carbons represent a rational geo-adaptive platform for mitigating pesticide residues in irrigation waters and food-processing streams associated with intensive rice-based production.

In South Asia, including India, additional biomass streams such as wheat straw and fruit-processing residues further contribute to a heterogeneous pool of potential carbon precursors [109,110]. While their composition can vary seasonally and regionally, these materials can yield carbons with mixed micro- and mesoporosity and moderate surface polarity [111,112,113,114], suitable for addressing the diverse pesticide profiles encountered in decentralized and small-scale food systems.

In North and South America, large-scale cultivation of cereals, oilseeds, and sugar crops generates substantial amounts of agricultural byproducts, including corn cobs, soybean residues, and sugarcane bagasse [115]. These biomass streams are closely associated with production systems in which herbicides represent the dominant class of applied pesticides, particularly glyphosate- and phenoxy-based formulations [116]. Carbon materials derived from cellulose-rich precursors such as sugarcane bagasse and corn residues can be tailored through activation strategies to yield porous structures with adjustable surface polarity [117,118]. Such features are especially relevant for the adsorption of highly polar or ionic herbicide molecules, for which purely graphitic or hydrophobic surfaces often exhibit limited affinity [119,120]. From a geo-adaptive perspective, these precursors provide flexible starting materials for developing adsorption systems that reflect the chemical reality of herbicide-intensive agricultural regions. In Brazil, where agricultural output is tightly coupled to both domestic food production and global export chains [121], the scale and continuity of biomass availability further support the feasibility of locally produced carbon-based adsorbents. The integration of bagasse- and crop-residue-derived carbons into water management and food-processing operations offers a context-specific pathway to reduce pesticide residues while aligning with sustainability and circular-economy objectives.

**Figure 5:**
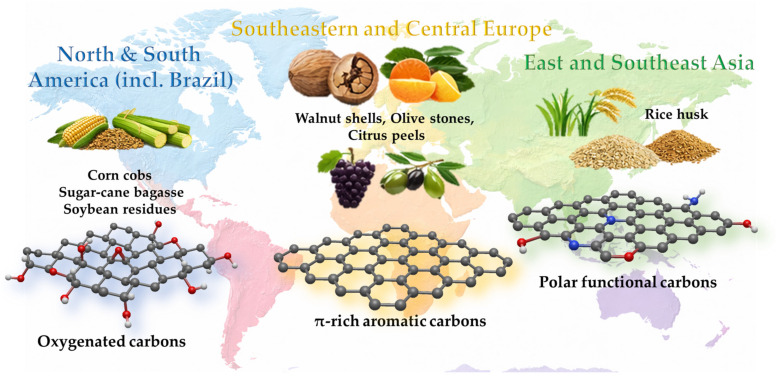
Locally available biomass and the types of carbon materials that are most commonly obtained from these sources.

Importantly, the use of regional biomass precursors contributes to sustainability beyond material performance alone. Local sourcing minimizes transportation-related emissions, enhances economic feasibility, and facilitates the decentralized production of adsorbents that can be directly integrated into food-processing or agricultural water-management systems. However, biomass variability arising from seasonal effects, cultivation practices, and processing conditions must be carefully considered to ensure reproducibility and consistent adsorption performance. Table 3 links regional biomass availability with carbon material features and pesticide classes relevant to food safety. Figure 5 graphically summarizes the locally most common biomass types and the types of carbon materials that can be obtained from these sources.

## 5. Towards Geo-Adaptive Food Safety Systems

The transition from a material-centered remediation toward geo-adaptive food safety systems requires moving beyond laboratory performance metrics and considering where, when, and how adsorption-based strategies can be realistically implemented along the food chain. In this context, geo-adaptive adsorbents are not envisioned as universal solutions, but as context-aware components integrated into specific processing steps, water streams, and product categories that reflect regional agricultural practices and regulatory constraints.

In many food production systems, pesticide residues enter processing lines through raw materials and associated wash waters rather than through direct application during processing [122]. Washing, blanching, soaking, and extraction steps represent potential control points for implementing adsorption-based mitigation strategies. Although experimental studies directly integrating adsorbents into food-processing environments remain limited [123], these stages offer conceptually attractive opportunities for the application of geo-adaptive adsorption materials to reduce pesticide residues. Geo-adaptive adsorbents can be selected or designed to match the dominant pesticide classes encountered at these stages, enhancing removal efficiency without requiring harsh processing conditions. For example, fruit and vegetable washing operations in fungicide- or organophosphate-dominated regions may benefit from adsorbents with accessible aromatic domains and moderate polarity, whereas herbicide-intensive systems linked to cereal and oilseed processing may require materials with a higher density of polar or charged surface sites. Importantly, adsorption systems integrated at these early stages can reduce pesticide loads before further processing, thereby lowering downstream contamination risks and improving overall food safety outcomes.

A key advantage of the geo-adaptive approach is its compatibility with decentralized and small-scale food systems. In many areas, especially in parts of Asia, South America, and the Mediterranean, food processing is performed by small or medium-sized businesses with limited access to centralized water treatment facilities [124]. Locally sourced biomass-based carbons provide a way to produce adsorbents that are both cost-effective and suited to local contamination issues. On-site adsorption units, integrated into irrigation reuse systems, washing lines, or effluent treatment steps, can deliver targeted cleanup without needing complex technology. By utilizing regionally abundant biomass and customizing material properties to local pesticide use patterns, these systems can enhance food safety in places where traditional treatment methods are difficult or too costly.

Beyond water treatment, geo-adaptive materials may also be used in food contact systems, including active packaging and contact filtration materials. While using carbon-based materials in direct contact with food requires careful consideration of migration, safety, and regulatory compliance, region-specific design principles can guide the development of materials that target locally relevant residues at trace levels. Instead of pursuing broadly adsorptive packaging materials, a geo-adaptive strategy focuses on selectivity and functionality tailored to specific food products and residue profiles. This approach is especially important for high-value fruits, juices, and minimally processed foods, where even small amounts of pesticide residues can have a disproportionate impact on regulation or consumer perception.

**Figure 6:**
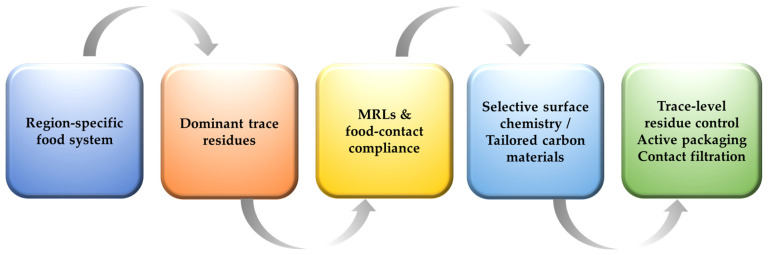
Geo-adaptive design aligns material selectivity with local residue profiles and regulatory constraints.

Any adsorption-based strategy deployed in food systems must comply with region-specific regulatory frameworks governing pesticide residues and food contact materials. Maximum residue limits, approved active substances, and food contact regulations differ substantially across regions, reinforcing the need for remediation strategies that are not only effective but also compliant. Geo-adaptive design offers a practical advantage in this respect by aligning material selection and performance targets with dominant local pesticide regulations. Instead of designing adsorbents to address globally banned or regionally irrelevant compounds, remediation efforts can focus on substances that are legally permitted yet problematic due to persistence, mobility, or cumulative exposure concerns. This alignment, schematically shown in Figure 6, increases the likelihood of regulatory acceptance and facilitates a smoother translation from laboratory research to real-world application.

Looking ahead, geo-adaptive food safety systems may incorporate responsive or modular adsorption strategies that evolve with changes in pesticide use, climatic conditions, and regulatory policies. As agricultural practices shift and new active substances are introduced or withdrawn, adsorption materials can be redesigned using the same regionally grounded principles rather than being replaced wholesale. Such adaptability emphasises the broader significance of geo-adaptive remediation as a systems-level concept. By connecting agricultural practices with locally available biomass, material design, and food safety requirements, geo-adaptive strategies provide a practical framework for reducing pesticide residues while remaining responsive to both global knowledge and local conditions.

## 6. Challenges, Limitations, and Future Perspectives

While the geo-adaptive design of carbon-based adsorbents offers a compelling framework for aligning pesticide remediation with regional food safety needs, several scientific, technical, and regulatory challenges must be acknowledged to ensure its responsible development and application. One of the primary limitations lies in the fragmented nature of available data on the relationships among pesticide occurrence, chemical speciation, and adsorption performance across regions. Although numerous studies report adsorption capacities for individual pesticides, these data are often generated under idealized laboratory conditions that do not reflect the complexity of real food matrices, variable water compositions, or region-specific processing environments. The absence of consistent databases that integrate pesticide use patterns, residue-monitoring data, and adsorption behavior remains a critical bottleneck to systematic geo-adaptive design.

Material reproducibility represents a second major challenge. Biomass-derived carbons inherently reflect the variability of their precursors, which are influenced by crop variety, growing conditions, seasonal factors, and processing history. While such variability underpins the geo-adaptive concept, it also complicates standardization and scale-up. Several practical strategies may help mitigate these limitations, including blending biomass batches to reduce compositional variability, optimizing carbonization or activation parameters to achieve more consistent structural properties, and post-synthesis surface modifications to tune adsorption behavior. Such approaches may improve the reproducibility and reliability of biomass-derived adsorbents while preserving the sustainability advantages of locally sourced precursors. Future research must therefore balance local adaptability with sufficient material consistency to meet food safety and regulatory expectations, potentially through controlled activation strategies or modular post-treatment approaches. From an application standpoint, translating geo-adaptive adsorbents into food systems requires careful evaluation beyond adsorption efficiency alone. Regeneration behavior, long-term stability, interactions with food components, and potential secondary contamination must be systematically assessed under food-relevant conditions. Importantly, materials intended for use in washing, filtration, or contact systems must be evaluated within existing food safety frameworks to ensure compliance with regulations governing food contact materials and residue limits. In many regulatory systems, materials that may come into contact with food products are subject to strict safety assessments addressing potential migration, toxicological risks, and long-term stability under processing conditions. Consequently, translating geo-adaptive adsorbents into food-related applications will require not only technical optimization but also careful consideration of regulatory approval pathways and risk assessment procedures.

Despite these challenges, the geo-adaptive framework offers several forward-looking opportunities. Rather than pursuing universally optimal materials, future research can adopt a design-for-context philosophy, where adsorbents are intentionally developed for specific combinations of crops, pesticides, climates, and processing practices. This shift encourages interdisciplinary collaboration among material scientists, food technologists, toxicologists, and regulatory experts, moving pesticide mitigation from a purely technical exercise toward an integrated food safety strategy.

In the longer term, geo-adaptive remediation concepts may support more resilient food systems capable of responding to evolving agricultural pressures and climate-driven changes in pesticide use. As regulatory landscapes shift and new active substances emerge, regionally grounded material design principles can be adapted without requiring the redesign of mitigation systems from first principles. In this sense, geo-adaptive adsorbents represent not a fixed technological solution, but a flexible framework for aligning material innovation with the dynamic realities of global food production.

Pesticide remediation in food systems should no longer be approached as a search for universally optimized adsorbents. Effective mitigation demands geo-adaptive strategies that explicitly align material design with region-specific agricultural practices, dominant pesticide chemistries, and food safety constraints. By reframing remediation as a locally informed, system-level challenge, and by directly linking agricultural realities, biomass valorization routes, adsorption mechanisms, and regulatory requirements, geo-adaptive design defines a clear and actionable pathway toward food safety strategies that are scientifically robust, practically relevant, and genuinely responsive to regional conditions of food production and consumption.

## 7. Conclusions

The concept of geo-adaptive design proposed in this review reframes pesticide remediation as a context-dependent challenge that must account for regional pesticide use patterns, environmental conditions, and locally available biomass resources. Rather than pursuing universally optimized adsorbents, this approach emphasizes the alignment between contaminant chemistry, adsorbent surface properties, and the agricultural systems in which pesticide residues occur. Within this framework, geo-adaptive carbon-based adsorbents can simultaneously address three key objectives relevant to pesticide mitigation in food systems. Efficiency is improved by matching adsorption mechanisms with the dominant pesticide classes encountered in specific agricultural regions. Selectivity arises from tailoring surface chemistry and pore structures to the molecular characteristics of target contaminants. At the same time, sustainability is enhanced by using locally abundant agro-industrial residues as carbon precursors, enabling decentralized material production while supporting circular-economy strategies within regional food systems. By integrating these dimensions, geo-adaptive design moves adsorption-based pesticide remediation beyond the search for universally optimal materials and toward solutions that are scientifically grounded, regionally relevant, and compatible with the realities of modern food production and processing.

## Figures and Tables

**Table 1:** Dominant pesticide groups and representative active substances by region [21,22,23,24,25,26].

Pesticide (Active Substance)	Pesticide Group	Main Crop Applications	USA	EU	China	India	Brazil	Comment—Regional Usage Pattern
Glyphosate	Herbicide (phosphonate)	Cereals (maize, wheat), soybean, oilseed crops, orchards, vineyards, non-selective weed control in crop fields	YES	YES (L)	YES	YES	YES	Dominant in the Americas and Brazil
2,4-D	Herbicide (phenoxy)	Cereals (wheat, maize), pasture and grassland systems, some fruit orchards	YES	YES	YES	YES	YES	Widely used in cereals (Americas, Asia)
Atrazine	Herbicide (triazine)	Maize, sorghum, sugarcane	YES	NO	YES	YES	YES	Banned in the EU; common in Brazil and the USA
Chlorpyrifos	Insecticide (organophosphate)	Fruits, vegetables, citrus, orchard crops, cereals	YES (L)	NO	YES	YES	YES	Still widely used in Asia and Latin America
Malathion	Insecticide (organophosphate)	Fruits, vegetables, citrus, stored grains	YES	YES	YES	YES	YES	Typical for fruit and vegetable production (warm regions)
Diazinon	Insecticide (organophosphate)	Fruits, vegetables, orchards, turf, and horticultural crops	NO	NO	YES	YES	YES	Phased out in the EU and USA; present elsewhere
Imidacloprid	Insecticide (neonicotinoid)	Vegetables, fruits, rice, potatoes, horticultural crops	YES	YES (SL)	YES	YES	YES	Dominant in Asia; restricted in the EU
Acetamiprid	Insecticide (neonicotinoid)	Fruits, vegetables, tea, citrus, horticultural crops	YES	YES	YES	YES	YES	One of the few neonicotinoids permitted in the EU
Carbaryl	Insecticide (carbamate)	Fruits, vegetables, orchards, cereals	YES	NO	YES	YES	YES	Legacy insecticide, still used outside the EU
Mancozeb	Fungicide (dithiocarbamate)	Fruits, vegetables, potatoes, vineyards	YES	YES	YES	YES	YES	Widely used in fruit and vegetable production
Chlorothalonil	Fungicide (chloronitrile)	Vegetables, potatoes, cereals, fruits	YES	NO	YES	YES	YES	Withdrawn in the EU; common elsewhere
Tebuconazole	Fungicide (triazole)	Cereals, vineyards, fruits	YES	YES	YES	YES	YES	Dominant in the EU and Brazil
Propiconazole	Fungicide (triazole)	Cereals, oilseed crops, fruits	YES	YES	YES	YES	YES	Similar to tebuconazole
Paraquat	Herbicide (bipyridylium)	Cereals, soybean, oilseed crops, plantation crops (e.g., banana, coffee), non-selective weed control	YES	NO	YES	YES	YES	Banned in the EU; common in Brazil and India

YES = approved/registered, frequently with limitations, NO = prohibited or not approved (status refers to the active substance, not all formulations), L = limited, SL = strictly limited.

**Table 2:** Dominant pesticide groups and representative active substances by regions [21,22,23,24,25,26].

Dominant Pesticide Class	Representative Active Substances	Regions Where It Is Commonly Used	Key Chemical Characteristics	Preferred Adsorbent Surface Features	Implications for Geo-Adaptive Design
Herbicides (phosphonate, phenoxy, triazine)	Glyphosate, 2,4-D, atrazine	USA, Brazil, parts of China, and India	Highly polar or ionic; limited aromaticity; strong hydration	Oxygen-containing functional groups; charged or polar surfaces; moderate porosity	Emphasis on surface functionality over hydrophobicity; adsorption driven by hydrogen bonding and electrostatic interactions
Insecticides (organophosphates)	Chlorpyrifos, malathion, diazinon	China, India, South America; legacy use in the EU	Moderate polarity; ester and phosphoryl groups; partial aromatic character	Mixed surface chemistry with both aromatic domains and polar sites	Balanced surface heterogeneity enables multiple interaction mechanisms
Insecticides (neonicotinoids)	Imidacloprid, acetamiprid	Asia, India; restricted use in the EU	Polar heterocycles; high water solubility	Polar surfaces with accessible functional groups; controlled pore accessibility	Targeting polar adsorption without excessive surface oxidation
Fungicides (triazoles, chlorinated)	Tebuconazole, propiconazole, chlorothalonil	EU, Mediterranean regions, Brazil	Aromatic rings; moderate hydrophobicity; high chemical stability	Extended π-conjugated domains; relatively hydrophobic graphitic surfaces	Preservation of aromatic character is critical; excessive oxidation may reduce affinity
Carbamate insecticides	Carbaryl	India, China, South America	Moderate polarity; aromatic backbone	Combination of π-rich domains and hydrogen-bond-active sites	Adsorbents should allow complementary interaction modes

**Table 3:** Regional biomass precursors and their relevance for geo-adaptive carbon-based adsorbents.

Region	Dominant Agricultural Byproducts	Key Biomass Characteristics	Typical Carbon Material Features	Target Pesticide Classes	Food System Relevance
Southeastern & Central Europe	Walnut shells, nut residues	High lignin content; low ash	Aromatic carbon frameworks; pronounced π-conjugation; moderate surface polarity	Organophosphate insecticides; triazole fungicides	Relevant for fruit, vegetable, and orchard production
Mediterranean Europe	Olive stones, olive pomace, grape pomace	Mixed lignocellulosic composition; residual lipids	Balanced micro/mesoporosity; moderately hydrophobic surfaces	Fungicides (triazoles, chlorinated); insecticides	Vineyard, olive oil, fruit, and vegetable chains
Mediterranean Europe	Citrus peels and pomace	Pectin- and cellulose-rich; high oxygen content	Oxygenated surfaces; moderate polarity; accessible porosity	Organophosphates; selected fungicides	Citrus fruit washing, juice, and beverage processing
East & Southeast Asia	Rice husks	High silica content; rigid structure	Hierarchical porosity; polar surface sites; enhanced accessibility	Neonicotinoids; carbamates; polar insecticides	Rice-based food processing and irrigation waters
North & South America (incl. Brazil)	Corn cobs, sugarcane bagasse, soybean residues	Cellulose-rich; variable mineral content	Tunable porosity after activation; adaptable surface functionality	Herbicides (glyphosate, phenoxy, triazines)	Large-scale cereal and oilseed processing
South & Southeast Asia	Rice straw, wheat straw, fruit pomace	Heterogeneous composition; seasonal variability	Mixed micro/mesoporosity; moderate polarity	Organophosphates; neonicotinoids	Diverse crops and decentralized food systems
Global	Fruit peels, juice pomace	High moisture; pectin-rich	Oxygenated surface groups; lower graphitization	Water-soluble pesticide residues	Juice, beverage, and fresh produce processing

## Data Availability

No new data were created or analyzed in this study. Data sharing is not applicable to this article.

## Abbreviations

The following abbreviations are used in this manuscript:

AGROFIT	Brazilian pesticide registration information system
ANVISA	National Health Surveillance Agency (Brazil)
CIBRC	Central Insecticides Board and Registration Committee (India)
EFSA	European Food Safety Authority
EPA	Environmental Protection Agency (United States)
FIFRA	Federal Insecticide, Fungicide, and Rodenticide Act (United States)
MAPA	Ministry of Agriculture, Livestock and Food Supply (Brazil)
MARA	Ministry of Agriculture and Rural Affairs (China)
MRL	Maximum Residue Limit
